# The Evolutionary History of Sarco(endo)plasmic Calcium ATPase (SERCA)

**DOI:** 10.1371/journal.pone.0052617

**Published:** 2012-12-20

**Authors:** Ianina Altshuler, James J. Vaillant, Sen Xu, Melania E. Cristescu

**Affiliations:** 1 Great Lakes Institute for Environmental Research, University of Windsor, Windsor, Ontario, Canada; 2 Department of Biology, Indiana University, Bloomington, Indiana, United States of America; 3 Department of Biology, McGill University, Montreal, Quebec, Canada; University of Ottawa, Canada

## Abstract

Investigating the phylogenetic relationships within physiologically essential gene families across a broad range of taxa can reveal the key gene duplication events underlying their family expansion and is thus important to functional genomics studies. P-Type II ATPases represent a large family of ATP powered transporters that move ions across cellular membranes and includes Na^+^/K^+^ transporters, H^+^/K^+^ transporters, and plasma membrane Ca^2+^ pumps. Here, we examine the evolutionary history of one such transporter, the Sarco(endo)plasmic reticulum calcium ATPase (SERCA), which maintains calcium homeostasis in the cell by actively pumping Ca^2+^ into the sarco(endo)plasmic reticulum. Our protein-based phylogenetic analyses across Eukaryotes revealed two monophyletic clades of SERCA proteins, one containing animals, fungi, and plants, and the other consisting of plants and protists. Our analyses suggest that the three known SERCA proteins in vertebrates arose through two major gene duplication events after the divergence from tunicates, but before the separation of fishes and tetrapods. In plants, we recovered two SERCA clades, one being the sister group to Metazoa and the other to Apicomplexa clade, suggesting an ancient duplication in an early eukaryotic ancestor, followed by subsequent loss of one copy in Opisthokonta, the other in protists, and retention of both in plants. We also report relatively recent and independent gene duplication events within invertebrate taxa including tunicates and the leech *Helobdella robusta*. Thus, it appears that both ancient and recent gene duplication events have played an important role in the evolution of this ubiquitous gene family across the eukaryotic domain.

## Introduction

A clear understanding of the evolutionary history of gene families is essential for studying their function, expression, and the evolutionary forces responsible for their diversification. Many evolutionary events such as gene duplication are important drivers of the expansion of gene families but can also confound functional genomics and gene expression studies that focus on orthologous genes. Consequently, a solid phylogeny of the genes of interest, especially multiple-copy genes, is needed before performing gene expression or comparative studies. The availability of many sequenced genomes greatly facilitates the investigation of the evolutionary history of many environmentally relevant gene families, such as the P-type II ATPases. This family of cation transporters plays a key role in the adaptation of organisms to variable environments, including variation in cation concentrations, due to their shared specificities for Ca^2+^, K^+^ and Na^+^ [1]. Although the nomenclature of this gene family has been revisited, it is generally accepted that P-type II ATPases include five closely related sub-families (SERCA, PMCA, NK/HK, ENA, and ACU) [1], [2], [3]. This study focuses on investigating the key evolutionary events that have led to the extensive diversification of sarco(endo)plasmic calcium ATPases (SERCA) across the major domains of eukaryotes.

Scarco(endo)plasmic Reticulum Calcium-ATPase (SERCA) is a key player in calcium signalling [4], which is involved in many aspects of cellular function [5], including transcription [6], cell motility [7], apoptosis, exocytosis, and signal transduction [8]. For example, during calcium-mediated signal transduction, the depolarization of the cell membrane in active cells causes an extensive influx of calcium into the cytoplasm. However, this influx of calcium needs to be reversed for proper cellular function [5]. To reduce cytoplasmic Ca^2+^ concentrations, SERCA uses ATP to actively pump calcium into the sarco(endo)plasmic reticulum for storage [4], [9]. The essential cellular function of SERCA makes it an interesting target for evolutionary studies as it is ubiquitous and indispensable across eukaryotic taxa.

Given the importance of the SERCA proteins to both cellular and organismal physiology, changes in the function, location, and expression of SERCA constitute significant evolutionary events. Previous genetic studies revealed that several gene duplication events occurred in the evolution of the *SERCA*. Three genes are present in vertebrates (*ATP2A1-3*), coding for three SERCA isoforms, SERCA 1-3 [9], while only one gene has been described in invertebrates, with the exception of the human parasitic blood fluke, *Schistosoma mansoni*, which has at least two [9], [10]. Interestingly, each of the vertebrate genes undergoes alternative splicing, resulting in ten SERCA proteins: SERCA 1a/b, SERCA 2a/b and SERCA 3a/b/c/d/e/f [11], [12]. These isoforms and their splice variants show a range of tissue specific expression patterns. For example, SERCA 1a is expressed in fast twitch muscles of adults and SERCA 1b in neonates [13]. SERCA 2a is expressed primarily in cardiac and slow-twitch skeletal muscles, whereas its splice variant, SERCA 2b, is expressed in almost all non-muscle cells and is often considered the house keeping variant [9], [12]. Furthermore, SERCAs 3 and 2b are found in a wide range of cells including lymphocytes, epithelial, endothelial, and mast cells, as well as Purkinje neurons of the cerebellum [9], [14]. The efficiency of the pump varies among the isoforms with SERCA 1a/b having a higher turnover rate than SERCA 2b and a higher affinity for calcium than SERCA 3 [14], [15]. Between the two SERCA 2 isoforms, SERCA 2b has a 2-fold higher calcium binding ability but a 2-fold lower turnover rate [14], [16]. The single SERCA gene in invertebrates also undergoes alternative splicing and shows tissue specific expression in a similar way to the vertebrate SERCA 2 [17], [18], [19]. SERCA 2 can be alternatively spliced to make a SERCA 2b (1042aa) protein or the shorter variant SERCA 2a (997aa), which has exons 22 and 24 spliced out compared with the longer protein [9], [20]. A similar pattern of splicing has been demonstrated in invertebrates such as *Artemia franciscana* and *Caenorhabditis elegans* [9], [17]. The single SERCA protein in invertebrates has been suggested to be most closely related to the vertebrate SERCA 2 based on the observation that SERCA2b is the housekeeping variant [9].

The P-type ATPases show a complex history of gene duplication events. For example, *Arabidopsis* has 46 known P-type ATPase genes with multiple isoforms in each family; comparatively humans have about 36 genes. Several hypotheses have been proposed to explain the evolution of this complex gene family. First, multiple isoforms could have evolved to be expressed in different cell types, and would have tissue specific regulation [21]. In this scenario, mutations in the promoter are important for isoforms to be expressed in distinctive amounts in different tissues or at different developmental stages [22]. Second, different isoforms could have evolved to function optimally under different cellular conditions or stressors (e.g., toxic cations) [23], allowing the organism to inhabit a wide variety of habitats and niches. In this case, mutations in coding sequence of the genes are most important, as they can alter the biochemical properties of the protein that are advantageous in specific environments. Lastly, it has been suggested that a fraction of isoforms are functionally redundant duplicates [21]. These alternative hypotheses are consistent with the theoretical predictions that the evolutionary fate of gene duplicates is difficult to distinguish [24]. To address this issue, an initial step is to characterize the historical gene duplication events that have occurred during the evolution of such gene families. SERCA is the most well characterized P-type ATPase, having X-ray crystallographic structures of its different conformational states and domains [25]. Despite the extensive knowledge and interest in its structure, function, and expression, little is known about SERCA’s evolutionary history. Here, we use protein sequences and phylogenetic reconstruction to examine the relationship among SERCA homologues across eukaryotic taxa. Specifically, we assess the role of gene duplication in the evolution of vertebrate SERCA isoforms and test previous hypotheses regarding the phylogenetic relationships of three vertebrate SERCA isoforms with invertebrate SERCA. Furthermore, we explore the protein-based eukaryotic phylogeny of SERCA to examine various likely gene duplication events in other phylogenetic lineages and their evolutionary implications.

## Materials and Methods

### Sequence Retrieval

A total of 81 SERCA amino acid sequences of vertebrate, invertebrate, plant, fungi, and other unicellular eukaryotes such as protists and ciliated protozoans were retrieved from Genbank, Uniprot, Ensembl, and JGI (Table S1). Sequences were chosen to span the known and confirmed SERCA genes across the eukaryotic kingdom. Preference was given to sequences that had protein level and transcript level evidence for the sequence over those inferred only from homology (Table S1). Searches were conducted using the key terms “Sarcoplasmic/endoplasmic calcium ATPase”, “SR Ca^2+^ ATPase” and “SERCA”. In addition, BLAST searches were conducted based on annotated sequences. If multiple splice variants of the protein were reported, the canonical sequence was used.

### Sequence Alignment and Phylogenetic Analyses

The amino acid sequence alignment was performed using ClustalW [26] with a gap opening penalty of 10 and gap extension penalty of 0.2. The highly variable 3′ terminal ends of the sequences were trimmed to avoid ambiguity. No other variable regions were excluded from the alignment. Phylogenetic analyses were performed using neighbour-joining (NJ) and Bayesian inference (BI) approaches, in MEGA [27] and MrBayes [28] respectively. Members of other P-type II ATPases sub-families were used as outgroups (Table S1) in all phylogenetic reconstructions. Outgroup sequences included the Plasma Membrane Ca^2+^ ATPase (*ATP2B1*), Secretory Pathway Ca^2^^+^ ATPase (*ATP2C1* and *ATP2C2*), Na^+^/K^+^-transporting ATPase (*ATP4A1*), K^+^-transporting ATPase (*ATP4A*), as well as the fungi-specific Na^+^/K^+^ ATPase (*ACU1*) and Na^+^ transport ATPase (*ENA1*) [29]. We used a Bayesian inference method for tree construction with the WAG model of amino acid substitution. This model was selected prior to the final analysis using a model jumping algorithm implemented in MrBayes [28], [30]. This algorithm regularly swaps between 9 different fixed-rate amino acid models throughout the analysis and selects the model with the highest contribution to posterior probability density [28], [30]. The WAG model can handle a large number of sequences and is applicable to a wide range of protein families, but retains the advantages of a maximum-likelihood approach and accounts for multiple substitutions at the same site [31]. Three independent runs of 4 Markov chains were conducted for 1,000,000 generations with a sampling frequency of 10 and the first 25% of sampled trees discarded as burn-in. We confirmed the topology of the Bayesian tree with a cluster based neighbour-joining tree using pairwise deletion and the Jones-Taylor-Thornton (JTT) amino acid model [32]. Node support was analyzed using 1000 bootstrap replicates.

## Results and Discussion

### Overall Phylogenetic Pattern

**Figure 1:**
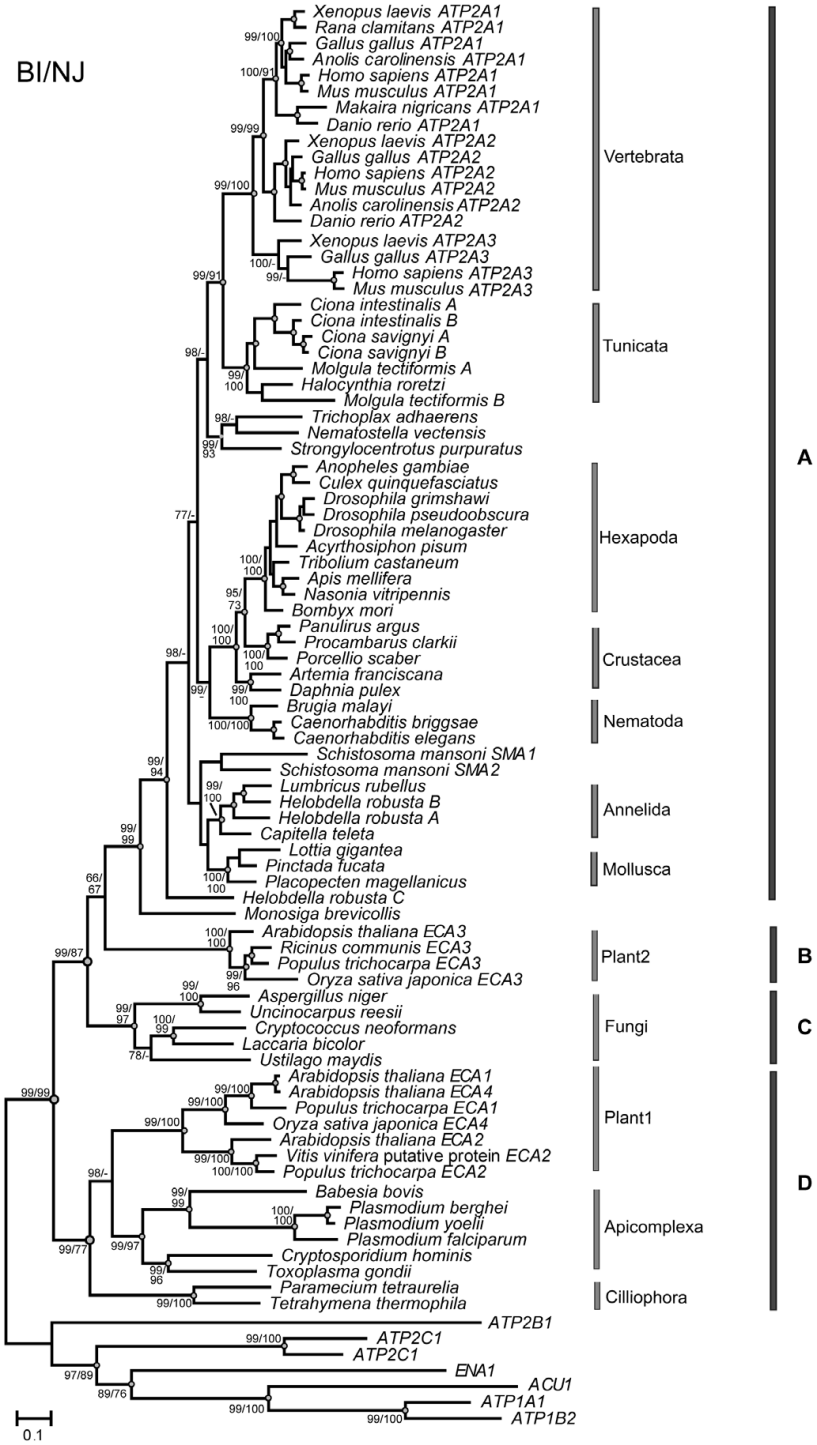
Bayesian phylogenetic reconstruction of SERCA amino acid sequences from 57 taxa. The numbers at the nodes indicate posterior probabilities/bootstrap supports. Nodes highlighted with gray circles represent consensus neighbouring-joining (NJ) and Bayesian Inference (BI) analyses with bootstrap support higher than 70%.

The SERCA alignment consisted of 81 sequences (61 unique taxa) spanning 1575 amino acids and contained 220 conserved and 818 parsimony-informative sites. Both the BI and NJ analyses returned highly congruent tree topologies. There were two major monophyletic clades. The first group contains clades A, B and C, which consist of metazoan, fungal, and plant sequences, respectively (Fig. 1). The second group contains clade D that encompasses plant and protist sequences (Fig. 1). Within clade A, the chordates are monophyletic and contain two reciprocally monophyletic clades corresponding to vertebrates and tunicates.

### SERCA Gene Duplication and Evolution

Within metazoans, the SERCA sequences of the chordates form a well supported monophyletic group that includes two sister clades, corresponding to the vertebrates and tunicates. In vertebrates, each of the three SERCA isoforms (i.e. SERCA1-3; coded by *ATP2A1-3*, respectively) form highly supported monophyletic groups. This pattern indicates that the three SERCA isoforms arose through two rounds of gene (or whole genome) duplication events after divergence from the tunicates but before the separation of fishes and tetrapods. Furthermore, *ATP2A1* and *2* are sister groups and likely arose from a more recent duplication event than the one leading to *ATP2A3*. Our findings do not support the previous suggestion that SERCA2 is the most ancestral of the three isoforms [9]. Furthermore, tunicates show evidence of several independent and relatively recent gene duplication events (Fig. 1).

Our study reveals that invertebrates also have experienced several instances of gene duplication events leading to multiple *SERCA* paralogs. Previous studies identified that the parasite trematode *Schistosoma mansoni* has two SERCA proteins that are coded by the genes *SMA1* and *SMA2* [10]. Based on our analysis, the two *S. mansoni* calcium ATPases group together, despite the low bootstrap support and high sequence divergence (29.5%). This suggests that *SMA1* and *2* are likely paralogs that arose from an ancient gene duplication event. Furthermore, *Helobdella robusta* also possesses three *SERCA* genes. While *SERCA A* and *B* group with other annelid sequences *SERCA C* is highly divergent and basal to the animal clade. Thus, *SERCA C* likely arose due to an ancient gene duplication event, while *SERCA A* and *B* are the result of a relatively recent gene duplication event. This is consistent with findings of several other gene duplications within the *H. robusta* genome, across a variety of unrelated gene families [33], [34], [35].

Plants are particularly reliant on calcium signalling to control many vital physiological processes and stress responses [36], [37]. Because plants use the Ca^2+^ to respond to different stimuli, the temporal and spatial patterns of the signals must vary [38]. Thus, the regulation of proteins involved in calcium signalling needs to be fine-tuned in order to induce a stimulus specific response [39]. While *Arabidopsis* is known to have four Endoplasmic Ca^2+^ ATPases (coded by genes *ECA 1-4*) [40], most plants have only three [21]. Based on our analysis, the plant sequences form two monophyletic clades, Plant1 and Plant2 (Fig. 1), consistent with previous findings in Viridiplantae [3]. Plant1 clade consists of the *ECA 1*, *2*, and *Arabidopsis ECA4* genes and is sister to the clade formed by apicomplexan sequences. However, Plant2, which contains the *ECA 3* genes, is sister to metazoan sequences. The presence of two divergent clades of *SERCA* genes in plants indicates a possible ancient duplication early in eukaryotic evolution followed by the loss of *ECA 3*-like protein from the protists and the loss of the other duplicate in the lineage leading to animals and fungi, whereas the plants retained both copies. Alternatively, lateral gene transfer (LGT) between protists and plants could have resulted in the Plant1 group. However, LGT events are rare and their prevalence in eukaryotes is unclear [41], [42]. Interestingly, the *ECA 3* encoded protein serves to pump calcium into both the Golgi and endoplasmic reticulum [43]. However, animals and fungi use a separate set of proteins (SPCA) to pump calcium into the Golgi that appear to be absent in plants [43]. The altered function and localization of the *ECA 3* encoded protein to the Golgi may have played a role in its evolution and maintenance in plant genomes and warrants further investigation. The evolution of complex calcium signalling in plants was likely facilitated by duplication of Ca^2+^ ATPase genes which diverged in their patterns of regulation and localization.

Gene duplication events are an essential part of the evolutionary process as they generate novel gene functions and families. The initial increased dosage of gene products resulting from a gene duplication event may be beneficial or detrimental to the organism. The function of the new gene will be retained through stabilizing selection if the increased dosage is beneficial or lost through purifying selection if it is detrimental [24]. However, if increased dosage has no effect, the gene is no longer under selective pressure and is free to accumulate mutations. Therefore, the duplicated gene can either become a pseudogene, or gain novel function through changes in the protein structure or expression pattern [44], [45]. Duplicated genes may also gain novel function by translocation into different regulatory regions. Such events can drastically alter the location, timing, and conditions of their expression. It appears that duplicated *SERCA* genes gain novel functions; this is especially apparent in the three vertebrate *SERCA* genes that exhibit tissue specific expression patterns likely resulting from divergence in regulation of these genes following the duplication events. Evidence of both ancient and recent gene duplication events in many taxa demonstrates the capacity of *SERCA* genes to multiply and retain functional significance.

### From Gene Tree to Species Tree: Paraphyly of Crustacea

If we ignore the major ancient gene duplication events, the overall phylogenetic pattern recovered was consistent with that of the combined protein data of α-tubulin, β-tubulin, actin, and elongation factor 1–alpha [46]. Moreover, the recovered phylogeny provides valuable information about the evolutionary path of crustaceans. The phylogenetic relationships among arthropod taxa, especially those within Pancrustacea, remain unclear in many phylogenetic studies [47]. Here, the monophyly of the Pancrustacea SERCA proteins is highly supported. The SERCAs of hexapods form a monophyletic group. However, crustaceans appear to be paraphyletic; *Panulirus argus*, *Procambarus clarkii*, and *Porcellio scaber* are sister to the hexapods, and not to the clade formed by the branchiopods *Daphnia pulex* and *Artemia franciscana*. This observation is consistent with other molecular and morphological based studies that support the monophyly of Pancrustacea, including all members Crustacea and Hexapoda [48], [49], [50], [51], [52], [53]. To date there is no consensus regarding the placement of Hexapoda within the paraphyletic crustacean group. Proposed sister clades include Branchiopoda [50], [51], Malacostraca [48], [49], and Copepoda [52]. Our SERCA protein-based phylogeny supports Malacostraca (lobsters, shrimp, woodlice) as the sister group to Hexapoda. However, future detailed studies based on a combination of morphological and molecular data are still necessary to elucidate the phylogenetic relationships within Pancrustacea.

### Conclusion

Overall, our phylogenetic analyses reveal several recent and ancient gene duplication events across different taxonomic levels during the evolution of *SERCA* genes. Notably, gene duplication events have resulted in proteins with new function and expression patterns in plants and vertebrates. Our results have refined the understanding of the complex evolutionary history of this gene family and will greatly facilitate gene expression and comparative studies that focus on *SERCA* genes.

## Supporting Information

Table S1 List of protein sequences used for phylogenetic analyses.
(DOCX)
