# Supplementary material for: The Evolutionary History of Sarco(endo)plasmic Calcium ATPase (SERCA)
Source: PLoS One. 2012 Dec 20;7(12):e52617. doi: 10.1371/journal.pone.0052617 (PMC3527596; doi:10.1371/journal.pone.0052617)
Supplement: Table S1 — List of protein sequences used for phylogenetic analyses. (DOCX) [file pone.0052617.s001.docx]

**Table S1**

| **Species** | **Common name** | **Paralogs** | **Accession number** | | **Evidence** |
| --- | --- | --- | --- | --- | --- |
| *Acyrthosiphon pisum* | pea aphid |  | GenBank ID: XP_001943129 | | EST |
| *Anolis carolinensis* | lizard | *ATP2A1* | Ensembl ID: ENSACAP00000005618 | | Ensemble genebuild prediction |
|  |  | *ATP2A2* | Ensembl ID: [ENSACAP00000015925](http://uswest.ensembl.org/Anolis_carolinensis/Transcript/ProteinSummary?db=core;g=ENSACAG00000016106;r=scaffold_123:2179386-2214386;t=ENSACAT00000016243) | | Ensemble genebuild prediction |
| *Anopheles gambiae* | African malaria mosquito |  | Uniprot ID: Q7PPA5 | | Inferred from homology |
| *Apis mellifera* | honey bee |  | GenBankID: XP_393851 | | EST |
| *Arabidopsis thaliana* | mouse-ear cress | *ECA1* | Uniprot ID: P92939 | | Protein level |
|  |  | *ECA2* | Uniprot ID: O23087 | | Transcript level |
|  |  | *ECA3* | Uniprot ID: Q9SY55 | | Transcript level |
|  |  | *ECA4* | Uniprot ID: Q9XES1 | | Transcript level |
| *Artemia franciscana* | brine shrimp |  | Uniprot ID: P35316 | | Transcript level |
| *Aspergillus niger* | fungi |  | Uniprot ID: A2RBD0 | | Inferred from homology |
| *Babesia bovis* | apicomplexan |  | Uniprot ID: A7AUB0 | | Inferred from homology |
| *Bombyx mori* | domestic silkworm |  | Uniprot ID: C7AQP4 | | Transcript level |
| *Brugia malayi* | nematode |  | Uniprot ID: A8PF97 | | Inferred from homology |
| *Caenorhabditis briggsae* | nematode |  | Uniprot ID: A8XSD9 | | Inferred from homology |
| *Caenorhabditis elegans* | nematode |  | Uniprot ID: Q9XTG6 | | Transcript level |
| *Capitella teleta* | polychaete worm |  | JGI Protein ID: 165811 * | | EST |
| *Ciona intestinalis* | sea squirt | A | JGI Protein ID: 207981 * | | EST |
|  |  | B | JGI Protein ID: 295594 * | | EST |
| *Ciona savignyi* | sea squirt | A | Uniprot ID: Q75UU1 | | Transcript level |
|  |  | B | Ensembl ID: [ENSCSAVP00000016616](http://uswest.ensembl.org/Ciona_savignyi/Transcript/ProteinSummary?db=core;g=ENSCSAVG00000009769;r=reftig_16:2728324-2733497;t=ENSCSAVT00000016797) | | EST |
| *Cryptococcus neoformans* | yeast |  | Uniprot ID: Q5KCV6 | | Inferred from homology |
| *Cryptosporidium hominis* | apicomplexan |  | Uniprot ID: Q5CNZ6 | | Inferred from homology |
| *Culex quinquefasciatus* | mosquito |  | Uniprot ID: B0XEW6 | | Inferred from homology |
| *Danio rerio* | zebrafish | *ATP2A1* | Uniprot ID: Q642Z0 | | Transcript level |
|  |  | *ATP2A2* | Uniprot ID: Q7ZW18 | | Transcript level |
| *Daphnia pulex* | water flea |  | JGI Protein ID: 219234 * | | EST |
| *Drosophila grimshawi* | fruit fly |  | Uniprot ID: GH20185 | | Inferred from homology |
| *Drosophila melanogaster* | fruit fly |  | Uniprot ID: P22700 | | Protein level |
| *Drosophila pseudoobscura* | fruit fly |  | Uniprot ID: Q292Q0 | | Inferred from homology |
| *Gallus gallus* | chicken | *ATP2A1* | Uniprot ID: P13585 | | Transcript level |
|  |  | *ATP2A2* | Uniprot ID: Q03669 | | Transcript level |
|  |  | *ATP2A3* | Uniprot ID: Q9YGL9 | | Transcript level |
| *Halocynthia roretzi* | tunicate |  | Uniprot ID: Q8IAC0 | | Transcript level |
| *Helobdella robusta* | leech | A | JGI Protein ID: 156619 * | | EST |
|  |  | B | JGI Protein ID: 106126 * | | EST |
|  |  | C | JGI Protein ID: 185062 * | | EST |
| *Homo sapiens* | human | *ATP2A1* | Uniprot ID: O14983 | | Protein level |
|  |  | *ATP2A2* | Uniprot ID: P16615 | | Protein level |
|  |  | *ATP2A3* | Uniprot ID: Q93084 | | Protein level |
| *Laccaria bicolor* | Mushroom |  | Uniprot ID: B0D3J7 | | Inferred from homology |
| *Lottia gigantea* | owl limpet |  | JGI Protein ID: 181748 * | | EST |
| *Lumbricus rubellus* | humus earthworm |  | Uniprot ID: D0EXD4 | | Transcript level |
| *Makaira nigricans* | Blue marlin |  | Uniprot ID: P70083 | | Transcript level |
| *Molgula tectiformis* | Tunicate | A | Uniprot ID: B5MGP1 | | Transcript level |
|  |  | B | Uniprot ID: B5MGP3 | | Transcript level |
| *Monosiga brevicollis* | choanoflagellate |  | JGI Protein ID: 7291 * | | EST |
| *Mus musculus* | mouse | *ATP2A1* | Uniprot ID: Q8R429 | | Transcript level |
|  |  | *ATP2A2* | Uniprot ID: O55143 | | Protein level |
|  |  | *ATP2A3* | Uniprot ID: Q64518 | | Transcript level |
| *Nasonia vitripennis* | jewel wasp |  | GenBank ID: XP_001603571 | | EST |
| *Nematostella vectensis* | sea anemone |  | JGI Protein ID: 160437 * | | EST |
| *Oryza sativa japonica* | rice | *ECA3* | Uniprot ID: Q10DF1 | | Inferred from homology |
|  |  | *ECA4* | Uniprot ID: Q8H8W1 | | Inferred from homology |
| *Panulirus argus* | Caribbean spiny lobster |  | Uniprot ID: Q49LV5 | | Transcript level |
| *Paramecium tetraurelia* |  |  | Uniprot ID: Q9N9D8 | | Transcript level |
| *Pinctada fucata* | pearl oyster |  | Uniprot ID: B2KKR1 | | Transcript level |
| *Placopecten magellanicus* | sea scallop |  | Uniprot ID: O77070 | | Transcript level |
| *Plasmodium berghei* | parasitic protist |  | Uniprot ID: Q4Z579 | | Inferred from homology |
| *Plasmodium falciparum* | parasitic protist |  | Uniprot ID: Q5R2K7 | | Inferred from homology |
| *Plasmodium yoelii* | parasitic protist |  | Uniprot ID: Q27764 | | Inferred from homology |
| *Populus trichocarpa* | poplar tree | *ECA1* | Uniprot ID: B9HPP7 | | Inferred from homology |
|  |  | *ECA2* | Uniprot ID: B9I912 | | Inferred from homology |
|  |  | *ECA3* | Uniprot ID: B9IBQ1 | | Inferred from homology |
| *Porcellio scaber* | woodlouse |  | Uniprot ID: Q8I897 | | Transcript level |
| *Procambarus clarkii* | red swamp crayfish |  | Uniprot ID: O17315 | | Transcript level |
| *Rana clamitans* | green frog | *ATP2A1* | Uniprot ID: Q9DDB9 | | Transcript level |
| *Ricinus communis* | castor oil plant | *ECA3* | Uniprot ID: B9R6Y5 | | Inferred from homology |
| *Schistosoma mansoni* | blood fluke | *SMA1* | Uniprot ID: Q27779 | | Transcript level |
|  |  | *SMA2* | Uniprot ID: O96527 | | Transcript level |
| *Strongylocentrotus purpuratus* | purple sea urchin |  | Uniprot ID: Q308S5 | | Transcript level |
| *Tetrahymena thermophila* | ciliate protozoa |  | Uniprot ID: Q22BT1 | | Inferred from homology |
| *Toxoplasma gondii* | apicomplexan |  | Uniprot ID: Q5IH90 | | Transcript level |
| *Tribolium castaneum* | red flour beetle |  | GenBank ID: XP_966783 | | EST |
| *Trichoplax adhaerens* | placozoan |  | Uniprot ID: B3S4L8 | | Inferred from homology |
| *Uncinocarpus reesii* | fungi |  | Uniprot ID: C4JXS5 | | Inferred from homology |
| *Ustilago maydis* | fungi |  | Uniprot ID: Q7Z8J8 | | Inferred from homology |
| *Vitis vinifera* | grape | *ECA2* | Uniprot ID: F6I6G6 | | Inferred from homology |
| *Xenopus laevis* | African clawed frog | *ATP2A1* | Uniprot ID: Q7ZXY6 | | Transcript level |
|  |  | *ATP2A2* | Uniprot ID: A2RV57 | | Transcript level |
|  |  | *ATP2A3* | Uniprot ID: Q0V9S4 | | Transcript level |
|  |  |  |  |  |  |
| **Outgroups** |  |  |  |  |  |
| *Homo sapiens* | human | ***ATP2B1*** | Uniprot ID: P20020 | | Protein level |
| *Homo sapiens* | human | ***ATP2C1*** | Uniprot ID: P98194 | | Protein level |
| *Homo sapiens* | human | ***ATP2C2*** | Uniprot ID: O75185 | | Protein level |
| *Homo sapiens* | human | *ATP1A1* | Uniprot ID: P05023 | | Protein level |
| *Homo sapiens* | human | *ATP4A* | Uniprot ID: P20648 | | Transcript level |
| *Ustilago maydis* | fungi | *ACU1* | Uniprot ID: Q703G5 | | Inferred from homology |
| *Saccharomyces cerevisiae* | fungi | *ENA1* | Uniprot ID: P13587 | | Protein level |

* All JGI Protein ID sequences were taken from their respective genome portal of the organism
